# Peptide Radioligands in Cancer Theranostics: Agonists and Antagonists

**DOI:** 10.3390/ph16050674

**Published:** 2023-04-30

**Authors:** Berthold A. Nock, Panagiotis Kanellopoulos, Lieke Joosten, Rosalba Mansi, Theodosia Maina

**Affiliations:** 1 Molecular Radiopharmacy, INRaSTES, NCSR “Demokritos”, 15310 Athens, Greece; nock_berthold.a@hotmail.com (B.A.N.); kanelospan@gmail.com (P.K.); 2 Department of Medical Imaging, Nuclear Medicine, Radboud University Medical Center, 6525 GA Nijmegen, The Netherlands; lieke.claessens-joosten@radboudumc.nl; 3 Division of Radiopharmaceutical Chemistry, Clinic of Radiology and Nuclear Medicine, University Hospital Basel, 4031 Basel, Switzerland; rosalba.mansi@usb.ch

**Keywords:** peptide theranostics, radiotheranostics, receptor antagonist, somatostatin radioligands, radiolabeled BBN-analogs, gastrin radioligands, exendin

## Abstract

The clinical success of radiolabeled somatostatin analogs in the diagnosis and therapy—“theranostics”—of tumors expressing the somatostatin subtype 2 receptor (SST_2_R) has paved the way for the development of a broader panel of peptide radioligands targeting different human tumors. This approach relies on the overexpression of other receptor-targets in different cancer types. In recent years, a shift in paradigm from internalizing agonists to antagonists has occurred. Thus, SST_2_R-antagonist radioligands were first shown to accumulate more efficiently in tumor lesions and clear faster from the background in animal models and patients. The switch to receptor antagonists was soon adopted in the field of radiolabeled bombesin (BBN). Unlike the stable cyclic octapeptides used in the case of somatostatin, BBN-like peptides are linear, fast to biodegradable and elicit adverse effects in the body. Thus, the advent of BBN-like antagonists provided an elegant way to obtain effective and safe radiotheranostics. Likewise, the pursuit of gastrin and exendin antagonist-based radioligands is advancing with exciting new outcomes on the horizon. In the present review, we discuss these developments with a focus on clinical results, commenting on challenges and opportunities for personalized treatment of cancer patients by means of state-of-the-art antagonist-based radiopharmaceuticals.

## 1. Introduction

**Figure 1:**
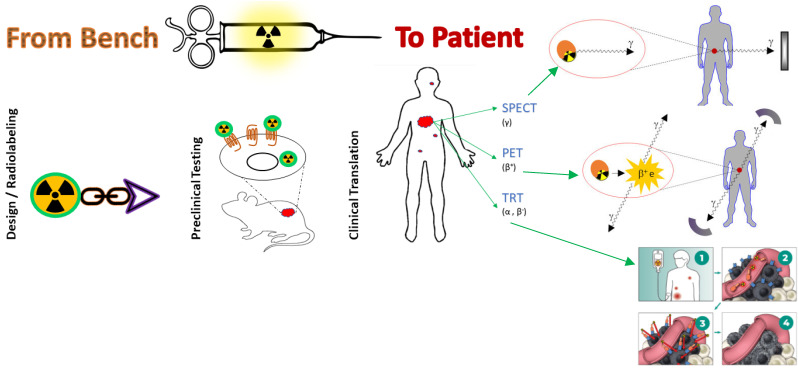
The development of a GPCR-directed theranostic peptide radioligand from bench to patient. The peptide conjugate is synthesized and radiolabeled and its biological profile evaluated first in cells and tumor-bearing mice expressing the target. Best candidates are selected for translation in humans. Labeling with a gamma emitter allows for SPECT imaging whereas a positron emitter enables PET imaging. Both techniques will indicate patients eligible for radiotherapy with an alpha, beta or Auger electron emitter to eradicate tumor cells, according to precision medicine principles.

The overexpression of G-protein coupled receptors (GPCRs) on the surface of cancer cells has provided the rationale for the development of radiolabeled peptides for diagnosis and therapy, the so-called “theranostics” of human cancer [1]. Depending upon the GPCR expressed in the particular cancer cell (Table 1), the respective native peptide ligand serves as a motif for the development of peptide analogs able to host and successfully deliver a diagnostic or therapeutic radionuclide to tumor lesions with high specificity [2]. Typically, a chelator is attached at the N-terminus of the peptide analog (directly or via a linker or spacer) for stable binding of the radiometal of choice. The latter is a gamma emitter for single photon emission computed tomography (SPECT; Tc-99m, In-111) or a positron emitter for positron emission tomography (PET; Ga-68, Cu-64) to allow for diagnosis and staging of primary or disseminated disease. Therapy can be applied based on diagnostic data by a beta (Lu-177, Y-90, Cu-67), alpha (Ac-225, Bi-213, Pb-212) or Auger electron (In-111) emitter to eradicate pathological lesions according to personalized medicine principles (Figure 1) [3,4].

The feasibility of this approach has been established with the advent of radiolabeled somatostatin analogs in the theranostic management of neuroendocrine tumor (NET) patients with a high expression of somatostatin subtype 2 receptors (SST_2_R) [5,6]. The pursuit for alternative radioligands for targeting a broader panel of GPCRs overexpressed on tumors has been thriving in the past few years, with breakthroughs achieved in many new areas, including the bombesin, gastrin and exendin derived radioligands discussed in this review (Table 1, highlighted in gray background) [1,7].

An exciting development in the field of somatostatin radioligands is a shift in paradigm from SST_2_R-agonist to antagonist radioligands, which were found to bind to more receptor sites on cancer cells, thereby compensating for their lack of internalization [8,9]. Several excised biopsy specimens, and tumor models in mice and patients revealed enhanced tumor values with radiolabeled antagonists [10]. In addition, the latter were cleared much more rapidly from the background [11]. Consequently, this new option was almost immediately investigated in the field of BBN-like radioligands, directed to the gastrin-releasing peptide receptor (GRPR) detected in many frequently occurring tumors, especially in prostate and mammary carcinoma [12]. This shift to GRPR-antagonists was particularly important for radiotherapy, in view of the adverse effects elicited by activation of the GRPR following systemic administration of potent BBN-like radioligands in higher amounts. Again, a fast background clearance could be achieved, even from organs physiologically expressing the GRPR [13]. Newest efforts toward antagonist-based radioligands to target the cholecystokinin subtype 2 receptor (CCK_2_R) [14] as well as the glucagon-like 1 receptor (GLP-1R) [15] have also been launched to overcome the problems of biosafety and elevated background. This review will present all of the above developments in the field for these four peptide ligand classes, illustrating the current status of research. Furthermore, limitations and challenges in the application of new radiolabeled antagonists in clinical routine will be emphasized along with exciting new prospects in the area of theranostic peptide radiopharmaceuticals.

## 2. Somatostatin

### 2.1. Somatostatin Its Receptors and NETs 

**Figure 2:**
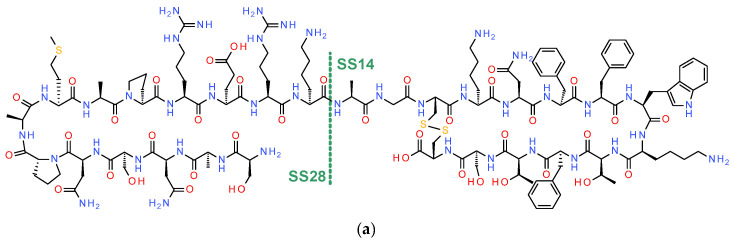

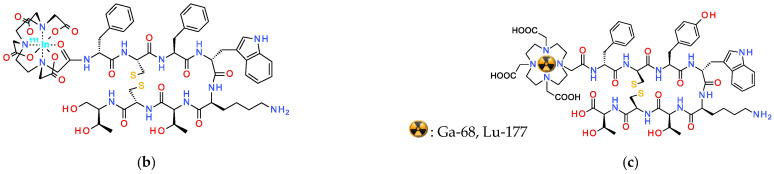
Chemical structures of (**a**) SS-28: H-Ser-Ala-Asn-Ser-Asn-Pro-Ala-Leu-Ala-Pro-Arg-Glu-Arg-Lys-Ala-Gly-c[Cys-Lys-Asn-Phe-Phe-Trp-Lys-Thr-Phe-Thr-Ser-Cys]-OH, and SS-14: H-Ala-Gly-c[Cys-Lys-Asn-Phe-Phe-Trp-Lys-Thr-Phe-Thr-Ser-Cys]-OH, (**b**) OctreoScan^®^: [^111^In]In-DTPA-DPhe-c[Cys-Phe-DTrp-Lys-Thr-Cys]-Thr(ol), and (**c**) [^68^Ga]Ga/[^177^Lu]Lu-DOTA-TATE: [^68^Ga]Ga/[^177^Lu]Lu-DOTA-DPhe-c[Cys-Phe-DTrp-Lys-Thr-Cys]-Thr-OH.

The high expression level of somatostatin receptors in human tumors, especially in NETs, as opposed to their lack of expression in most physiological tissues, has provided the molecular basis for theranostics with the aid of radiolabeled somatostatin analogs [1]. In fact, peptide receptor radionuclide therapy (PRRT) of NETs, guided by PET/CT results, applying suitably designed somatostatin radioligands represents a most successful paradigm of the theranostic approach in nuclear medicine [16]. Five somatostatin receptor subtypes (SST_1_R-SST_5_R) have been identified in humans, belonging to the GPCR family. These subtypes differ mainly in the extracellular and intracellular ends and are differently distributed in healthy tissues and in cancer [1,17,18]. Ligand-induced activation of any of SST_1_R-SST_5_R stimulates multiple intracellular cascades, eventually modulating neuronal activity, growth hormone release, or insulin, gastric acid and glucagon secretion [19]. It should be noted that the SST_2_R is the most clinically relevant subtype because it is most frequently expressed in NETs and neuroendocrine neoplasms (NENs) [20]. The natural somatostatin is present either as SS-14, consisting of 14 amino acids 12 of which form a ring, and representing the primary form in the brain, or as SS-28, containing 28 amino acids (the SS-14 elongated at the N terminus by a 14 amino acid chain) and representing the primary form of the hormone in the gut (Figure 2). 

Both forms are rapidly degraded in vivo by peptidases, especially neutral endopeptidase (NEP), a factor limiting their clinical application prospects [21]. Structure activity relationships studies have yielded synthetic octapeptide analogs with a six-member ring, which were resistant to proteolytic degradation, but typically losing their pan-somatostatin character, becoming SST_2_R-preferring. For example, octreotide (OC, DPhe-c[Cys-Phe-DTrp-Lys-Thr-Cys]-Thr(ol)) and lanreotide (Lan, D-2-Nal-c[Cys-Tyr-DTrp-Lys-Val-Cys]-Thr-NH_2_) are sufficiently stable and SST_2_R-affine to be successfully used in the treatment of advanced NETs [22,23]. Moreover, the introduction of suitable chelators at their N-terminus has allowed radiolabeling with medically relevant radiometals. As a result, attractive radioligands were developed showing strong in vivo targeting of SST_2_R-positive tumors and favorable pharmacokinetics in animals and in humans. In addition, radiopharmaceuticals with high clinical impact in the management of NETs have been approved and are currently routinely used (vide infra). 

The abovementioned developments have significantly upgraded the theranostic arsenal of anti-NET drugs with new powerful molecular tools. It should be noted that in recent decades, the incidence of NETs has constantly increased. These tumors are neoplasms arising from cells of the endocrine system in many organs in the body (gastrointestinal tract, lung or pancreas). Their diagnosis is often delayed due to their slow-growing nature and the lack of early symptoms [24].

### 2.2. Radiolabeled Somatostatin in Theranostics of NETs—Clinical Impact

Nowadays, the use of radiopharmaceuticals based on somatostatin agonists play a pivotal role in the management of NET patients [16,25,26]. Radioactivity was first introduced on a [Tyr^3^]OC (TOC) via radioiodination (I-123) of the Tyr^3^-residue and the resulting radioligand established for the first time in humans the feasibility of visualizing endocrine-related tumors [27]. Coupling of suitable chelators at the N-terminus of octreotide and its analogs, such as DTPA (diethylenetriaminepentaacetic acid), has allowed labeling with clinically interesting metallic radionuclides. Thus, in the early nineties OctreoScan^®^ ([^111^In]In-DTPA-OC, In-111 pentetreotide; Figure 2) was approved by the US Food and Drug Administration (FDA) and was the first peptide radiopharmaceutical used in clinical applications for the diagnosis and staging of NETs applying conventional gamma cameras or SPECT/CT [6]. In view of the excellent nuclear, logistic and imaging qualities of Tc-99m, a few analogs carrying either HYNIC/EDDA (HYNIC, hydrazinonicotinamide; EDDA, ethylenediaminediacetic acid) [28], or an open tetraamine (N_4_, 1,4,8,11-tetraazaundecane) at the N-terminal DPhe were developed [29,30] and tested in NET patients applying SPECT and SPECT/CT thereafter, leading to higher sensitivity compared with OctreoScan^®^. For theranostic applications though the demand for SST_2_R–radioligands-based positron emitters for PET and PET/CT and therapeutic beta-emitters (e.g., Y-90 and Lu-177) drove research toward analogs functionalized with the universal chelator DOTA (1,4,7,10-tetraazacyclododecane-1,4,7,10-tetraacetic acid), or other chelators, suitable for stable labeling with trivalent radiometals of medical interest (Figure 2), thereby revolutionizing the field [31].

Accordingly, a plethora of somatostatin analogs were developed with optimized qualities, including receptor affinity, specificity, internalization rates and/or pharmacokinetics. The most widely used are DOTA-TOC and DOTA-TATE (TATE, [Thr^8^-OH]TOC; Figure 2). Freeze-dried formulations (kits) for DOTA-TATE (NETSPOT^®^) and DOTA-TOC (SomaKit TOC^®^) have been approved by the FDA and the European Medicines Agency (EMA) for labeling with the PET radiometal Ga-68 [9,32]. A systematic review and meta-analysis reported the clinical impact of Ga-68-SS PET/CT on the management of patients with NETs [33]. On more than 1500 patients included in this study, a change in patient management occurred in 44% of the cases, concluding that PET/CT using [^68^Ga]Ga-DOTA-TATE/TOC/NOC(NOC, [1-Nal^3^]OC) is vital for patient management. Most importantly, Ga-68-SS PET/CT serves as a predictive biomarker to confirm expression of the receptor-target and identify patients suitable for PRRT with [^177^Lu]Lu-DOTA-TATE, or a similar therapeutic counterpart. Recently, newer somatostatin radioligands have been developed for PET/CT, bearing optimized radiometal-chelates. For Ga-68 the bifunctional hybrid chelator DATA^5m^ ((6-pentanoic acid)-6-(amino)methyl-1,4-diazepinetriacetate) was coupled on DPhe^1^ of TOC, allowing for easy access to ready-to-use [^68^Ga]Ga-DATA^5m^-TOC in the clinic [34]. In a comparative study in 50 patients with biopsy-proven GEP-NETs, [^68^Ga]Ga-DATA-TOC PET/CT showed a similar diagnostic efficacy to that of [^68^Ga]Ga-DOTA-NOC. Thus, [^68^Ga]Ga-DATA-TOC was determined to be an effective and safe alternative to [^68^Ga]Ga-DOTA-NOC with the added benefit of ease, cost-effective and improved yield of instant kit-type synthesis. In another approach, the bifunctional chelator MECOSar (5-(8-methyl-3,6,10,13,16,19-hexaaza-bicyclo[6.6.6]icosan-1-ylamino)-5-oxopentanoic acid) was conjugated with TATE (SAR-TATE), allowing for stable binding of radiocopper (Cu-64/Cu-67) and effectively overcoming the limitations of DOTA for coordination of radiocopper, especially those related with in vivo stability [35]. Following promising preclinical results, [^64^Cu]Cu-SAR-TATE was tested for the first time in humans in 10 NEN patients, showing higher lesion uptake and retention and high-contrast images up to 24 h compared to [^68^Ga]Ga-DOTA-TATE [36]. A clinical trial with the therapeutic counterpart [^67^Cu]Cu-SAR-TATE is currently ongoing with the aim of evaluating the safety and efficacy of this new radiopharmaceutical in pediatric patients (NCT04023331). On the other hand, [^18^F]AlF-NOTA-OC ([^18^F]AlF-OC; NOTA, 2,2′,2″-(1,4,7-triazacyclononane-1,4,7-triyl)triacetic acid) has emerged as a new candidate for PET diagnosis of NETs [37]. Notably, a robust and automated labeling process has been established facilitating clinical use. [^18^F]AlF-OC was tested in a prospective, multicenter trial in comparison with Ga-68-SS PET/CT in NET patients (NCT04552847), showing that [^18^F]AlF-OC outperformed [^68^Ga]Ga-DOTA-TATE/NOC on PET/CT, thereby validating its use in clinical practice [37].

In the PRRT arena, [^177^Lu]Lu-DOTA-TATE ([^177^Lu]Lu-oxodotreotide) received marketing authorization for the treatment of patients with SS-positive gastroentropancreatic NETs (GEP-NETs) according to the NETTER-1 clinical trial (NCT01578239) results. In this study, a significant improved progression-free survival (PFS) was confirmed in advanced progressive midgut NET patients after four cycles of [^177^Lu]Lu-DOTA-TATE (4 × 7.4 GBq) plus a low dose (30 mg) long-acting release OC (PRRT group, n = 117), as compared to patients treated with high-dose (60 mg double dose) long-acting release OC (control group, n = 114) [38,39]. However, five years after the last patient randomization no statistically significant difference was observed in the median overall survival (OS) between the [^177^Lu]Lu-DOTA-TATE arm and the control arm (11.7 months), despite a clinically significant improvement in the quality of life and PFS in the [^177^Lu]Lu-DOTA-TATE arm. Most importantly, the NETTER-1 study revealed a low incidence of long-term side effects, including hematotoxicity and nephrotoxicity [40]. Further evidence from a meta-analysis study, integrating several clinical data, contributed to the inclusion of [^177^Lu]Lu-DOTA-TATE in the therapeutic planning as an effective and safe treatment option for NETs [41]. Interestingly, recent data from the retrospective NETTER-R study conducted on 110 patients with pan NETs revealed improved survival in patients who received no chemotherapy prior to [^177^Lu]Lu-DOTA-TATE treatment, indicating a potential benefit of PRRT as an early treatment option [42]. At present, a NETTER-2 trial (NCT03972488) is ongoing with the aim of determining whether the combination of [^177^Lu]Lu-DOTATATE with long-acting OC prolongs PFS in grade-2/3 GEP-NETs as first-line treatment. All of the above studies shed light on the importance of PRRT in the management of NET patients and will help to further clarify this treatment’s position compared to other systemic therapies.

Today, the interest in radiopharmaceuticals based on somatostatin agonists is still high, a fact illustrated by the advent of alpha emitters in recent years, such as Bi-213, Ac-225, or Pb-212. Targeted alpha therapy (TAT) is expected to be more effective than PRRT with β^−^ emitters owing to the higher linear energy transfer (LET) of alphas, effectively inducing clustered double-strand DNA breaks and consequently rapid cell death. Although the short range of alpha particles (50–100 μm, i.e., approximately two or three cell diameters) limits the effective range of treatment, it is also responsible for less damage to surrounding healthy tissue. In a first-in-human alpha therapy study, the efficacy of the new radiopharmaceutical [^213^Bi]Bi-DOTA-TOC was reported in seven patients with metastatic NET, refractory to OC and progressing under β^−^ treatment with [^90^Y]Y/[^177^Lu]Lu-DOTA-TOC [43]. Notably, TAT was shown to induce tumor remission with acceptable acute and mid-term toxicity at therapeutically effective doses. It should be noted, that the therapeutic application of the short-lived Bi-213 (46 min) in the clinic is hampered both by the high cost and the currently still restricted supply of high activity [^225^Ac]Ac/[^213^Bi]Bi-generators. As a result, the clinical use of Ac-225 is favored taking into account its longer half-life (9.9 d) and its decay chain; the latter involves the generation of multiple alpha particles associated with higher cytotoxicity. Both DOTA-TOC and DOTA-TATE have been labeled with Ac-225 and therapy studies have been conducted in NET patients as an end-of-line treatment option [44,45]. [^225^Ac]Ac-DOTA-TOC was administered in 39 patients with progressive NEN, to estimate the most appropriate single cycle and cumulative doses in terms of hematological and renal toxicity. The analysis showed that repeated doses of ~20 MBq in 4-month intervals and a cumulative dose of 60-80 MBq were hematologically tolerable, without high grade (3/4) hematotoxicity, although data on chronic nephrotoxicity were not conclusive due to pre-existing risk factors [44]. A prospective single-arm study conducted on 32 patients with metastatic GEP NETs demonstrated that [^225^Ac]Ac-DOTA-TATE therapy effectively improves quality of life, inducing high response rates with a low toxicity profile [45]. Quite recently, a phase 1 clinical trial with [^212^Pb]Pb-DOTAM-TATE (DOTAM, 2,2′,2″,2‴-(1,4,7,10-tetraazacyclododecane-1,4,7,10-tetrayl)tetraacetamide; AlphaMedix^TM^) TAT in 20 NET patients (NCT03466216) was initiated and is currently ongoing. Preliminary results suggest a well-tolerated treatment with an overall response rate of 80% in the first 10 subjects treated at the dose of 2.50 MBq/kg (67.6 μCi/kg) for each cycle [46].

### 2.3. Shift in Paradigm: From Radiolabeled SST_2_R-Agonists to Antagonists

In recent decades, the search for radiolabeled somatostatin receptor antagonists for clinical application has been growing. Established SST_2_R-agonists, such as DOTA-TATE, have served as motifs for the design of the corresponding SST_2_R-antagonists, leading to theranostic agents with attractive profiles in cell and animal models compared to agonists. Thus, most radiolabeled SST_2_R-antagonists developed in recent years, despite their inability to internalize in target cells, recognize and strongly interact with a higher number of SST_2_R on the cell surface of target cells (comprising both active and inactive forms of the receptor). As a result, they display high and prolonged tumor uptake combined with a fast background clearance translating into excellent tumor-to-background ratios [47,48,49,50].

**Figure 3:**
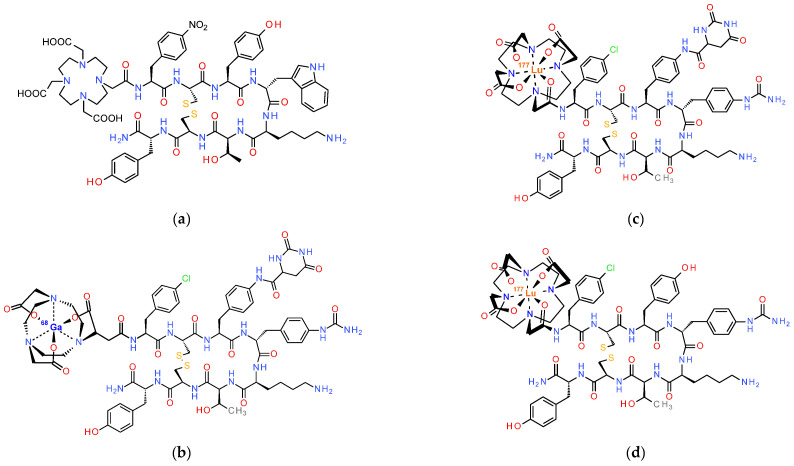
Chemical structures of (**a**) DOTA-BASS: DOTA-pNO_2_-Phe-c[DCys-Tyr-DTrp-Lys-Thr-Cys]-DTyr-NH_2_, (**b**) [^177^Lu]Lu-DOTA-JR11, or [^177^Lu]Lu-OPS201: DOTA-pCl-Phe-c[DCys-Aph(Hor)-DAph(Cbm)-Lys-Thr-Cys]-DTyr-NH_2_, (**c**) [^68^Ga]Ga-NODAGA-JR11, or [^68^Ga]Ga-OPS202, and (**d**) [^177^Lu]Lu-DOTA-LM3: [^177^Lu]Lu-DOTA-pCl-Phe-c[DCys-Tyr-DAph(Cbm)-Lys-Thr-Cys]-DTyr-NH_2_.

The first report on the superior performance of radiolabeled SST_2_R-antagonists compared to agonists, involved the preclinical evaluation of [^111^In]In-DOTA-BASS (BASS,-pNO_2_-Phe-c[DCys-Tyr-DTrp-Lys-Thr-Cys]-DTyr-NH_2_; Figure 3), and was found to be a game changer in the field [11]. These exciting results were next confirmed in the clinic via a prospective study including five NET or thyroid cancer patients. Notably, SPECT/CT established a higher tumor detection rate for [^111^In]In-DOTA-BASS (25/28 lesions) than OctreoScan^®^ (17/28 lesions) in a lesion-based analysis [51]. In the search for further improved SST_2_R-antagonists, structure–activity relationship studies have identified JR11 (pCl-Phe-c[DCys-Aph(Hor)-DAph(Cbm)-Lys-Thr-Cys]-DTyr-NH_2_; Aph(Hor), 4-amino-L-hydroorotyl-Phe; DAph(Cbm), D-4-(carbamoyl)amino-Phe) and LM3 (pCl-Phe-c[DCys-Tyr-DAph(Cbm)-Lys-Thr-Cys]-DTyr-NH_2_; Figure 3) as the most interesting candidates [52]. These synthetic analogs were found to be highly sensitive to the radiometal-chelate attached at their N-terminus [53]. Comprehensive studies with JR11 and LM3 in combination with different chelators and radiometals concluded that DOTA is the chelator of choice for In-111, Lu-177 and Y-90, while for Ga-68 NODAGA (1,4,7-triazacyclononane,1-glutaric acid-4,7-acetic acid) seems to be the most appropriate. Remarkably, the [^68^Ga]Ga-DOTA-analogs lost affinity for SST_2_R [53,54], with [^68^Ga]Ga-DOTA-JR11 having an affinity 40 times lower compared to [^177^Lu]Lu-DOTA-JR11 (29 ± 2.7 nM vs. 0.7 ± 0.15 nM, respectively). On the other hand, [^68^Ga]Ga-NODAGA-JR11 showed 24 times higher affinity than [^68^Ga]Ga-DOTA-JR11. This discrepancy led to the decision of using two different radiopharmaceuticals as theranostic pair. Accordingly, [^68^Ga]Ga-NODAGA-JR11 (known as [^68^Ga]Ga-OPS202) is applied for diagnosis and [^177^Lu]Lu-DOTA-JR11 (known as [^177^Lu]Lu-OPS201) for treatment of NETs. In line with these observations, the hybrid chelators DATA^5m^ and AAZTA^5^ (1,4-bis(carboxymethyl)-6-[bis(carboxymethyl)]amino-6-[pentanoic-acid]perhydro-1,4-diazepine) have been recently coupled to the SST_2_R-antagonist LM4 ([4Pal^3^]LM3; 4Pal, (4-pyridyl)Ala), allowing easy and stable coordination of Ga-68 and Lu-177, respectively, convenient in a clinical setting. Preliminary results in a small number of NET patients seem promising and warrant further validation [55,56].

The preclinical and clinical evaluation of radiolabeled somatostatin antagonists has been always accompanied by direct comparison with state-of-the-art radiolabeled somatostatin agonists. In a prospective phase I/II imaging study, [^68^Ga]Ga-OPS202 has been compared in 12 patients to [^68^Ga]Ga-DOTA-TOC. [^68^Ga]Ga-OPS202 presents lower uptake in gastrointestinal tract and increased tumor-to-background ratios with improved imaging contrast for liver metastases resulting in a useful tool for detecting primary GEP-NETs [57,58]. In a comparative preclinical study, the [^177^Lu]Lu-OPS201 antagonist showed superior tumor uptake and longer tumor retention in SST_2_R-expressing xenografts compared to [^177^Lu]Lu-DOTA-TATE. Furthermore, in a following pilot clinical study involving four NET patients, [^177^Lu]Lu-OPS201 showed 1.7 to 10.6 times higher tumor doses compared to [^177^Lu]Lu-DOTA-TATE. Moreover, the 6.2- and 7.2-times-higher tumor-to-kidney and tumor-to-bone marrow dose ratios represent an improvement in efficacy and toxicity of this treatment [59]. These results provided the impetus for a subsequent clinical study on the therapeutic application of [^177^Lu]Lu-OPS201 involving 20 NET patients [60], reporting an impressive overall response rate of 45%. However, patients with an estimated bone marrow dose ≥1.44 Gy experienced G4 thrombocytopenia (and G3/4 neutropenia) and 57% developed G4 myelosuppression after the second cycle, although no hematotoxicity was evident in patients with ≤1.08 Gy bone marrow dose. Consequently, the study was suspended and the therapeutic protocol was revised to reduce the bone marrow dose and halve the therapeutic dose in the second cycle. Interestingly, a substantial reduction in abdominal accumulation of [^177^Lu]Lu-OPS201 could be achieved by increasing the administered peptide amount without compromising tumor uptake in animal models [61]. Based on these findings, a mass escalation phase 1/2, international, multicenter, open-label PRRT trial clinical trial (NCT02592707) was initiated to evaluate the therapeutic efficacy following variations of administered activity and peptide amount. The study is concluded and although results are not published yet, an interim report claimed a disease control rate of 90% (95% CI: 68.3–98.8) at 12 months for the 20 NET patients with an adequate follow-up.

**Figure 4:**
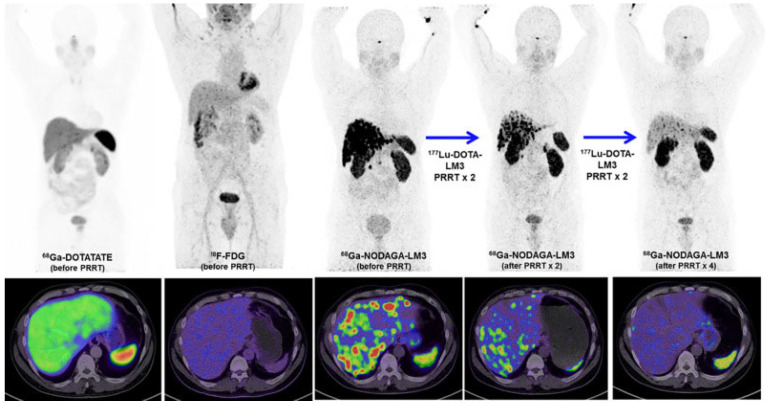
A patient with well-differentiated, non-functioning metastatic pancreatic NEN. [^68^Ga]Ga-DOTA-TATE PET/CT showed lesions in liver and lymph nodes with extremely low uptake (leftmost image), not exhibiting significant glucose hypermetabolism (second image from left). [^68^Ga]Ga-NODAGA-LM3 PET/CT instead showed disseminated metastases, with intense uptake in liver and lymph nodes (third image from left). After four cycles of [^177^Lu]Lu-DOTA-LM3 PRRT, restaging [^68^Ga]Ga-NODAGA-LM3 PET/CT showed excellent response (partial remission, rightmost image).This research was originally published in *JNM* 2020 ([64]; https://jnm.snmjournals.org/content/jnumed/62/11/1571/F6.large.jpg).

At the same time, the SST_2_R-antagonist LM3 has also been developed for clinical application with [^68^Ga]Ga-DOTA/NODAGA-LM3 and [^177^Lu]Lu-DOTA-LM3 applied as an alternative theranostic pair. In a prospective, randomized, double-blind, phase I/II, single-center study conducted on 16 NET patients [^68^Ga]Ga-NODAGA-LM3 and [^68^Ga]Ga-DOTA-LM3 showed favorable biodistribution with a high tumor uptake and retention, resulting in high image contrast [62]. Following this work, a prospective randomized, double-blind study (NCT04318561) was conducted on 40 patients with well differentiated NETs and results confirmed the superior diagnostic efficacy of PET/CT with [^68^Ga]Ga-NODAGA-LM3 compared to [^68^Ga]Ga-DOTA-TATE [63]. [^68^Ga]Ga-NODAGA-LM3 PET/CT imaging served as predictive biomarker for treatment with [^177^Lu]Lu-DOTA-LM3 in a compassionate-use study (Figure 4), including 51 patients with grade 1–3 metastatic NENs [64]. A significant therapeutic efficacy of [^177^Lu]Lu-DOTA-LM3 could be reported with a partial response and DCR (RECIST 1.1 criteria in 47 patients) of 36% and 85% at 3–6 months, respectively. The treatment was well-tolerated with thrombocytopenia occurring in only a few patients (maximal grade 3 thrombocytopenia in 5.9% of patients).

In addition to the theranostic Ga-68/Lu-177 radionuclide pair, other radionuclide candidates have recently been emerging as potential theranostic tools, associated with better treatment prospects and expected to dynamically address the problem of tumor radioresistance. Especially of note, radioisotopes of terbium (Tb-149/152/155/161) seem quite promising in this respect. For example, Tb-161 showing a similar decay pattern to Lu-177 additionally emits a substantial number of conversion and Auger electrons [65]. In this regard, a preclinical therapy study was conducted on SST_2_R-expressing xenografts comparing [^161^Tb]Tb-DOTA-TOC and [^161^Tb]Tb-DOTA-LM3 with their Lu-177 counterparts. [^161^Tb]Tb-DOTA-LM3 resulted in higher survival rates compared both to [^161^Tb]Tb-DOTA-TOC and to [^177^Lu]Lu-DOTA-LM3 [66]. These data supported the initiation of a new clinical trial (NCT05359146) aiming to measure the therapeutic index of [^161^Tb]Tb-DOTA-LM3 vs. [^177^Lu]Lu-DOTA-TOC in the same GEP-NET patients.

### 2.4. Conclusions

Somatostatin radioligands have attracted the attention of researchers from a broad spectrum of disciplines for more than 30 years and can be duly considered the driving force of peptide radiopharmaceutical development. The advent of theranostic somatostatin radiopharmaceuticals in clinical practice has undoubtedly improved the quality of life and survival of NET patients. Above all expectations, the field is continuously evolving to competently address a number of challenges and make available novel improved molecular tools and modalities [67]. Firstly, the shift in paradigm from internalizing SST_2_R-agonists to antagonists has been based on the ability of the latter to bind to considerably higher numbers of target sites on tumors [10,49]. Hence, radiolabeled antagonists may be applied for broader clinical indications, including tumors characterized by relatively low SST_2_R expression, such as renal cell carcinoma, small-cell lung cancer, breast cancer, non-Hodgkin lymphomas, medullary thyroid cancer, pheochromocytoma and paraganglioma. Secondly, the development of somatostatin radioligands binding to SSTR subtypes beyond the SST_2_R could address the issue of SST_2_R is downregulation or loss, reported in advanced disease stages with a worse prognosis. These analogs could be administered as a “cocktail” or as multi-targeting ligands. In the latter case, pharmacokinetics have to be carefully monitored. Multi-targeting radioligands can be developed to target not only SSTR subtypes, but different GPCRs co-expressed in human cancer. This approach is expected to tackle problems, such as target-receptor heterogeneity of expression, tumor resistance to drug/radiotherapies and loss of specific receptor subtypes during disease progression [68].

Finally, a number of exciting innovative approaches to enhance the efficacy of current PRRT to eradicate tumors have recently been proposed and are currently under investigation as PRRT adjuvant modalities [69]. For example, PRRT in combination with a poly(ADP-ribose) polymerase-1 (PARP)-inhibitor, checking the DNA repair mechanism, was shown to prolong tumor growth-inhibition and improve the median survival in a preclinical model compared to PRRT alone [70]. Moreover, the use of epigenetic drugs, such as DNA methyltransferase inhibitors (DNMTis) and histone deacetylase inhibitors (HDACis), and their involvement in the regulation of SSTRs and SST expression in NETs has been extensively discussed in a recent review [71]. A deeper understanding of the epigenetic mechanisms may be key to develop or improve treatment options for NET patients with sub-optimal SSTR expression levels.

## 3. Bombesin—Gastrin-Releasing Peptide

### 3.1. Bombesin/Gastrin-Releasing Peptide and Their Receptors in Human Tumors

**Figure 5:**
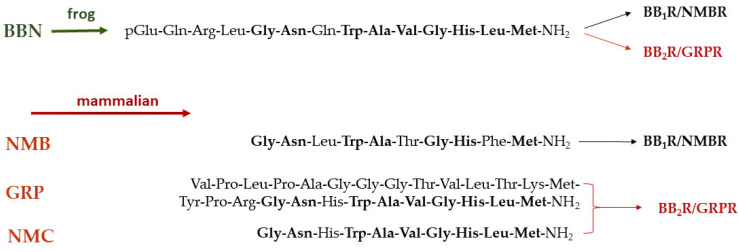
Amino acid sequences of frog BBN, mammalian NMB, GRP and NMC, with conserved residues highlighted in bold; each peptide preferably binds to the receptor subtype(s) indicated by the arrow(s) on the right-hand side of the diagram.

Bombesin (BBN) is a linear tetradecapeptide first isolated from the skin of the European frog *Bombina bombina* [13,72,73]. In recent years, BBN and its analogs have attracted much attention in the fields of oncology and nuclear medicine owing to their ability to interact with gastrin-releasing peptide receptors (GRPR) overexpressed in human tumors. It is well-known that BBN recognizes two major GPCRs located on the cell membrane of target cells. These receptors can be pharmacologically distinguished by their different binding affinity to native mammalian peptide analogs of amphibian BBN. The first is the neuromedin B receptor (NMBR), otherwise known as bombesin receptor 1 (BB_1_R), with high binding affinity for neuromedin B (NMB). The second is the GRPR, also referred to as bombesin receptor 2 (BB_2_R), with high binding affinity for the 27mer gastrin-releasing peptide (GRP) and its C-terminal decapeptide fragment neuromedin C (NMC, GRP(17-27)) [72]. The above frog BBN and the native mammalian peptides NMB, GRP and NMC, are characterized by high homology (Figure 5). It should be noted that a third bombesin receptor, BB_3_R, has been additionally discovered, an orphan receptor with no native ligand identified yet. The above receptors are physiologically expressed in mammalian organs and tissues, such as the pancreas (GRPR-positive), stomach (GRPR-positive) and intestines (GRPR- and NMBR-positive).

Most importantly, the GRPR is expressed in high numbers in a variety of malignant tumors [74], including the frequently occurring prostate [75,76,77] and breast cancer [78,79,80,81], small cell lung cancer [82], gastrinoma, gastrointestinal stromal tumors [83] and others [84]. During recent years, properly tailored BBN-like peptides have been proposed as theranostic anti-cancer agents, capable of delivering diagnostic (gamma/positron emitting) radionuclides or therapeutic payloads (beta^−^ and alpha particle emitters) to GRPR-expressing tumor lesions. In this way, GRPR-targeted diagnosis (applying SPECT or PET imaging technology) and therapy become feasible in an integrated approach, abiding to current personalized medicine principles. Interestingly, a good number of promising anti-GRPR radioligands have already entered clinical studies, drawing the attention of the pharmaceutical industry [13,85,86].

At this point, it is important to underline basic differences between the somatostatin and the BBN system. Firstly, biosafety issues following intravenous (iv) injection of either peptide are markedly different [87]. Unlike somatostatin, exerting inhibitory actions on target cells, BBN-like peptides are mitogenic and exert stimulatory actions on target-cells [88,89]. Hence, a series of adverse effects are inadvertently elicited in the body, the severity of which relies both on the dose and the potency of the compound administered. For example, BBN-induced activation of GRPRs in the gastrointestinal tract is known to stimulate bowel movement and secretion of gut hormones [90,91]. A second point to consider is the metabolic stability discrepancy between the in vivo robust cyclic octapeptides used in somatostatin-radiopharmaceutical development (e.g., octreotide and derivatives thereof) and the linear BBN-like peptide radioligands. The latter undergo rapid degradation on their way to target-cells by omnipresent peptidases, among which neutral endopeptidase (neprilysin, NEP) has been shown to play a major role [92,93]. Last but not least, dissimilar pharmacokinetic profiles arising from different distribution patterns of somatostatin or bombesin receptors, but also from dissimilar excretion pathways or clearance rates between somatostatin and BBN radioligands, directly translate to separate dosimetric outcomes and concerns. As a result of such inherent differences, the course of development of the respective receptor agonist/antagonist-based radioligands has been distinct between somatostatin and BBN.

### 3.2. Radiolabeled BBN Analogs in Cancer Theranostics: Limitations in Clinical Translation

A great number of BBN-like radiopeptides have emerged in the past few decades [13,85]. The majority of these compounds are based on the C-terminal nona-/octapeptide fragment BBN(6/7-14), preserving full binding affinity for the GRPR. In addition, full-chain BBN as well as GRP and C-terminal fragments thereof (e.g., NMC) have also served as motifs in anti-GRPR radioligand development [94,95]. In most cases, a suitable bifunctional chelator is covalently attached at the N-terminus of these motifs either directly or via a variety of linkers to allow for stable binding of clinically appealing radiometals, similar to those used in the case of somatostatin analogs (see Sections 1 and 2.2). The linker, on the other hand, may serve several purposes, such as to adjust pharmacokinetics, fine-tune affinity to the receptor, improve metabolic stability, or enable the attachment of extra functionalities (e.g., dyes for optical/fluorescent imaging, a second targeting moiety, etc.) [96]. Many of these variations have been pursued to date, resulting in a plethora of new analogs to target GRPR-expressing tumors [13].

It should be noted that these GRPR-agonist-based radiopeptides typically display high internalization rates in cancer cell lines, such as in human androgen-independent prostate adenocarcinoma PC-3 cells or human breast T-47D cells broadly used as GRPR-positive cell models. Xenografts as well as orthotopic tumors thereof have been raised in animals to study the profile of new compounds in vivo as well. These studies have revealed shortcomings in the performance of many BBN-like radioligands. For example, hepatobiliary excretion together with inadvertent targeting of GRPR-sites on intestinal walls has often led to high abdominal radioactivity levels, requiring careful radioligand design to enhance renal excretion without negatively affecting receptor affinity or internalization rates [97,98,99]. Moreover, high pancreatic uptake and retention causes dosimetry concerns in the case of radionuclide therapy with particle emitters. Another problem arises from the sub-optimal metabolic stability of the new radiopeptides, especially against NEP, compromising their tumor-targeting efficacy [92,93]. Administration of NEP-inhibitors (e.g., phosphoramidon (PA), thiorphan, or Entresto), or use of homo-/heterodimers have been proposed as potential means to address this problem [93,100,101].

**Figure 6:**
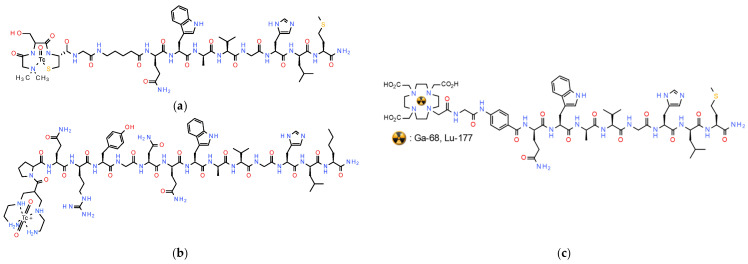
Chemical structures of (**a**) [^99m^Tc]Tc-RP527: [^99m^Tc]Tc-N_3_S-Gly-5Ava-Gln-Trp-Ala-Val-Gly-His-Leu-Met-NH_2_, (**b**) [^99m^Tc]Tc-DB4: [^99m^Tc]Tc-N_4_-Pro-Gln-Arg-Tyr-Gly-Asn-Gln-Trp-Ala-Val-Gly-His-Leu-Nle-NH_2_, and (**c**) [^68^Ga]Ga/[^177^Lu]Lu-AMBA: [^68^Ga]Ga/[^177^Lu]Lu-DOTA-Gly-4-aminobenzoyl-Gln-Trp-Ala-Val-Gly-His-Leu-Met-NH_2_, showing the metal chelate, linker and amino acid sequence; 5aVa: 5-aminovaleric acid.

In view of the above, only a handful of GRPR-agonist-based radioligands have shown promising results in the clinic, predominantly in prostate cancer patients and to a lesser extent in breast and other cancer patients, as previously reported. The first proof-of-principle studies with [^99m^Tc]Tc-RP527 ([^99m^Tc]Tc-N_3_S-Gly-5aVa-BBN(7-14), 5aVa: 5-aminovaleric acid; Figure 6) in a small number of metastasized prostate and breast cancer patients applying SPECT established the feasibility of GRPR-targeted diagnostic imaging of these tumors and their metastases. These studies also revealed the unfavorable pharmacokinetics of [^99m^Tc]Tc-RP527, showing high hepatobiliary excretion and hence high abdominal background [97]. These drawbacks were efficiently addressed with the introduction of [^99m^Tc]Tc-DB4 ([^99m^Tc]Tc-N_4_-[Pro^1^,Nle^14^]BBN(7-14); Figure 6), whereby an acyclic tetraamine chelator (1,4,8,11-tetraazaundecane via a carboxy anchor at position 6) was covalently coupled to Pro^1^ of full-length BBN, having the oxidation-susceptible Met^14^ replaced by Nle^14^ [98]. This modification resulted in a notably enhanced renal excretion and minimization of abdominal values both in animal models and in humans, attributed to the formation of the monocationic hydrophilic octahedral *trans*-[[^99m^Tc]Tc^V^(O)_2_(N_4_)]^+^-complex instead of the neutral lipophilic square pyramidal [^99m^Tc]Tc^V^O(N_3_S)-complex of [^99m^Tc]Tc-RP527 (Figure 6). In a group of prostate cancer patients, [^99m^Tc]Tc-DB4 was well-tolerated and effectively visualized pathological lesions in primary prostate cancer patients on SPECT/CT. However, in more advanced hormone-refractory prostate cancer with bone involvement, it was not as successful, calling attention to the impact of changes of GRPR-expression levels at different stages of the disease on diagnostic accuracy [102].

Aiming toward a theranostic application of BBN-like radioligands, the universal chelator DOTA was coupled to the BBN(7-14) motif via a Gly-4-aminobenzoyl linker giving rise to AMBA (DOTA-Gly-4-aminobenzoyl-BBN(7-14); Figure 6). AMBA could be labeled with Ga-68 for diagnostic imaging with PET/CT [103], as well as with Lu-177 for radionuclide therapy [88]. In fact, [^177^Lu]Lu-AMBA was first introduced as a candidate radiopharmaceutical in the treatment of androgen-refractory prostate cancer in men [99]. In preclinical studies, [^177^Lu]Lu-AMBA displayed high internalization rates in PC-3 cells and good uptake in PC-3 tumors in mice, but, at the same time, sub-optimal metabolic stability and unfavorably high abdominal values [92,99]. Most disappointingly, the clinical evaluation of [^177^Lu]Lu-AMBA in prostate cancer patients was terminated because of the severe adverse effects observed after iv injection of the (radio)ligand [88].

### 3.3. Biosafety Concerns: Switching to Radiolabeled GRPR-Antagonists

Indeed, serious biosafety concerns have become evident following the clinical translation of the GRPR-agonist AMBA, ranging from moderate abdominal discomfort and tachycardia after administration of diagnostic doses of [^68^Ga]Ga-AMBA [103] to severe manifestations throughout the gastrointestinal system when therapeutic doses of [^177^Lu]Lu-AMBA were injected to patients [88]. These effects were induced by activation of the GRPR physiologically expressed in the body and especially in the gastrointestinal tract [87]. Hence, the biosafety challenges revealed during the clinical translation of AMBA provoked a shift in paradigm in the field of BBN-radioligands from GRPR-agonists to antagonists with the goal to acquire clinically safe analogs [12]. In principal, antagonists do not activate their cognate receptor upon binding; however, these are known to compete with agonists for receptor binding, preventing agonist-induced receptor activation. At the same time, GRPR-antagonists do not internalize in target cells [104].

Numerous GRPR-antagonists available from past decades have served as motifs in the design of radiolabeled analogs with clinical efficacy and safety [104]. GRPR-antagonists were earlier developed as molecular tools in the elucidation of GRPR pharmacology, but also as anticancer therapies via inhibition of the mitogenic effects of BBN-like ligands. Most of these molecules are based on [DPhe^7^]BBN(7-14), whereby the C-terminal Leu^13^-Met^14^-NH_2_ dipeptide has undergone structural modifications, such as amino acid substitutions (e.g., to Sta^13^-Leu^14^-NH_2_, Sta: (3S,4S)-4-amino-3-hydroxy-6-methylheptanoic acid) [105], truncation of Met^14^ and alkylamidation/esterification of the exposed carboxy group of Leu^13^ [106], truncation of the dipeptide altogether and alkylamidation/esterification of the exposed carboxy group of His^12^ [107], to name a few. Attachment of the appropriate chelator at the N-terminus of these motifs, either directly or via a linker, has led to a plethora of analogs suitable for labeling with clinically interesting radiometals. Extensive structure-activity relationship (SAR) studies have been conducted in GRPR-expressing cells and tumor-bearing animals, with PC-3 cells and PC-3 xenografts in mice prevailing as a model. A few common characteristics could be established during these preclinical studies, for example, the inability of the radiolabeled GRPR-antagonists to internalize in cancer cells, their higher in vivo metabolic stability compared with agonists, as well as their faster clearance from the background (even from GRPR-rich organs such as the pancreas) than from tumors, overall displaying much more favorable pharmacokinetic profiles than agonists [87]. Pharmacokinetics could be further modified via selection of metal-chelate and/or linker, by administration of protease inhibitors, or by scaling up the amount of administered peptide dose, an option prohibited for agonists due to biosafety restrictions [13].

**Figure 7:**
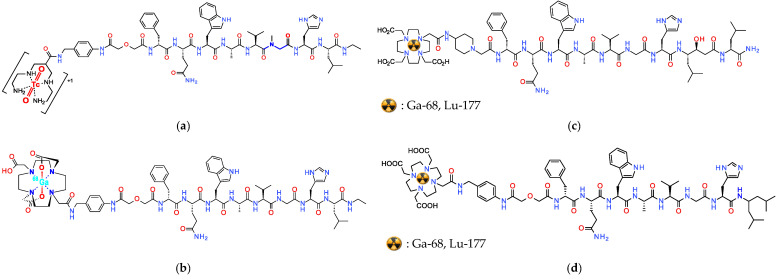
Chemical structures of (**a**) [^99m^Tc]Tc-DB15: [^99m^Tc]Tc-N_4_-AMA-DGA-[DPhe^6^,Sar^11^,Leu^13^-NHEt]BBN(6-13), (**b**) [^68^Ga]Ga-SB3: [^68^Ga]Ga-DOTA-AMA-DGA-[Dphe^6^,Leu^13^-NHEt]-BBN(6-13), (**c**) [^68^Ga]Ga/[^177^Lu]Lu-RM2: [^68^Ga]Ga/[^177^Lu]Lu-DOTA-Pip-[Dphe^6^,Sta^13^,Leu^14^-NH_2_]BBN(6-14), and (**d**) [^68^Ga]Ga/[^177^Lu]Lu-NeoBOMB1: [^68^Ga]Ga/[^177^Lu]Lu-DOTA-AMA-DGA-[Dphe^6^,His^12^-NHCH(CH_2_-CH(CH_3_)_2_)_2_]BBN(6-12), showing the metal chelate, linker and amino acid sequence.

Such extensive design and preclinical screening of radiolabeled GRPR-antagonists has brought to light candidates eligible for clinical translation, mostly performed in prostate and breast cancer patients. Hence, the efficacy and safety of radiolabeled GRPR-antagonists were explored in the clinical arena for diagnostic imaging (with SPECT or PET), and radionuclide therapy. In a proof-of-principle study in advanced breast cancer patients [^99m^Tc]Tc-DB15 ([^99m^Tc]Tc-N_4_-AMA-DGA-[Dphe^6^,Sar^11^,Leu^13^-NHEt]BBN(6-13), AMA: *p*-aminomethylaniline, DGA: diglycolic acid; Figure 7) was excellently tolerated and was able to visualize several metastatic lesions, both in the skeleton and in soft tissues [108]. Likewise, encouraging results with [^68^Ga]Ga-SB3 ([^68^Ga]Ga-DOTA-AMA-DGA-[Dphe^6^,Leu^13^-NHEt]-BBN(6-13); Figure 7) were first acquired with PET/CT in patients with disseminated prostate and breast cancer [109]. Pathological lesions were visualized in approximately 50% of the patients, despite their advanced disease and previous therapies. In a following study in a small number of therapy-naïve prostate cancer patients [^68^Ga]Ga-SB3 showed high diagnostic sensitivity and excellent correlation with GRPR-status in primary cancer lesions [110]. This result once again emphasized the impact of GRPR-expression status during the evolution of the disease and preceding treatments on diagnostic accuracy [13]. Unexpectedly, therapy options with the respective therapeutic [^177^Lu]Lu-SB3 analog were compromised by the observed rapid degradation of circulating radioligand by NEP, revealing prominent effects of radiometal-chelate on stability and pharmacokinetics [93].

A second group of radiolabeled GRPR-antagonists were based on the [Dphe^6^,Sta^13^,Leu^14^-NH_2_]BBN(6-14) motif [105], with a number of analogs tested in a small number of prostate and breast cancer patients in combination with SPECT or PET. Amongst those, [^68^Ga]Ga-RM2 ([^68^Ga]Ga-DOTA-Pip-[Dphe^6^,Sta^13^,Leu^14^-NH_2_]BBN(6-14), Pip: 4-amino-1-carboxymethyl-piperidine; Figure 7) holds a prominent place [111,112] because it has been investigated as the diagnostic member of a theranostic pair completed by the therapeutic counterpart [^177^Lu]Lu-RM2 [113]. In a group of 15 biopsy-confirmed cases of primary breast carcinoma, [^68^Ga]Ga-RM2 visualized 13 out of the 18 lesions on PET/CT, with a strong correlation of imaging results with estrogen receptor expression in primary tumors of untreated patients (ClinicalTrials.gov Identifier: NCT03831711) [111]. The profile of the PET-tracer in different groups of prostate cancer patients was reported in a number of studies. Of particular interest is a study of [^68^Ga]Ga-RM2-PET/MRI conducted in 32 prostate cancer patients with biochemical recurrence of the disease and negative findings on conventional imaging (ClinicalTrials.gov Identifier: NCT02624518). [^68^Ga]Ga-RM2 PET identified recurrent disease in 23 of the 32 participants, whereas the simultaneous MRI scan identified findings compatible with recurrent prostate cancer in only 11 of the 32 patients [112]. Interestingly, results from the first in-human dosimetry study with [^177^Lu]Lu-RM2 in a group of 35 metastatic castration-resistant prostate cancer patients was recently reported [113]. The therapy was well-tolerated and no side-effects were observed (average injected activity 4.5 ± 0.9 GBq). The radioligand showed high tumor uptake and rapid clearance from normal organs, with absorbed doses in tumor lesions being therapeutically relevant. Rapid and increased uptake was observed in the pancreas, in soft tissue and bone, whereby the main dose limiting organ was the pancreas.

**Figure 8:**
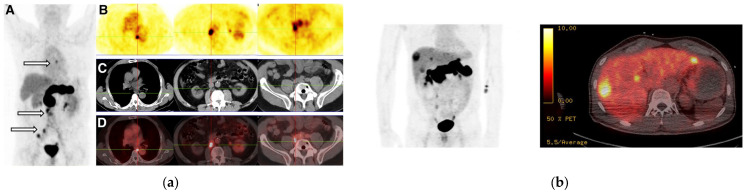
[^68^Ga]Ga-NeoBOMB1 PET/CT in (**a**) in a prostate adenocarcinoma patient, postradical prostatovesiculectomy with pelvic lymphadenectomy, intensity-modulated radiotherapy, and androgen-deprivation therapy: PET MIP (**A**), serial PET transverse (**B**), corresponding CT transverse (**C**), and fusion PET/CT (**D**) images. Multiple mediastinal, abdominal, paraesophageal, and pelvic lymph node metastases are indicated by arrows and crossbars.—This research was originally published in *JNM* 2017 (Ref. [114]; https://jnm.snmjournals.org/content/jnumed/58/1/75/F6.large.jpg). (**b**) a patient with GIST of ileum and histologically verified liver metastases (left: PET MIP, right: transverse fusion PET/CT)—This research was originally published in *JNM* 2020 ([117]; https://jnm.snmjournals.org/content/jnumed/61/12/1749/F4.large.jpg).

Of particular interest is the theranostic pair [^68^Ga]Ga/[^177^Lu]Lu-NeoBOMB1, generated by the coupling of DOTA at the N-terminus of the [Dphe^6^,His^12^-NHCH(CH_2_CH(CH_3_)_2_)_2_]-BBN(6-12) motif via an AMA-DIG (AMA: *p*-aminomethylaniline, DGA: diglycolic acid; Figure 7) linker, thus enabling labeling with medical radiometals, such as Ga-68 (for PET) and Lu-177 (for therapy) [114]. In fact, alkylamide derivatives of human [(N-acetyl)His^20^,His^25^-NHR]GRP(20-25), and especially when R = NHCH(CH_2_CH(CH_3_)_2_)_2_, represent a successful class of very potent GRPR-antagonists, characterized by high receptor affinity and in vivo enzymatic stability [107]. This sequence corresponds to the amphibian [Dphe^6^,His^12^-NHCH(CH_2_CH(CH_3_)_2_)_2_]BBN(6-12) motif used in NeoBOMB1. During preclinical evaluation [^68^Ga]Ga/[^177^Lu]Lu-NeoBOMB1 showed high receptor affinity and GRPR-specific cell-uptake, high metabolic stability and high and prolonged tumor uptake in GRPR-expressing tumors in mice [114,115,116]. These promising results were subsequently confirmed in a first-in-man study with [^68^Ga]Ga-NeoBOMB1 PET/CT in four prostate cancer patients. The radioligand was excellently tolerated and achieved clear visualization of primary prostate cancer and metastatic lesions in the lymph nodes, bone and soft tissue [114]. In line with this, multiple mediastinal, abdominal, paraesophageal, and pelvic lymph node metastases were successfully visualized in a prostate adenocarcinoma patient, following postradical prostatovesiculectomy with pelvic lymphadenectomy, intensity-modulated radiotherapy, and androgen-deprivation therapy on [^68^Ga]Ga-NeoBOMB1 PET/CT, as shown in Figure 8. Similar findings were obtained from a following phase I/Iia clinical trial (EudraCT 2016-002053-38) in nine patients with advanced GIST, with a representative ileal GIST patient showing strong uptake of [^68^Ga]Ga-NeoBOMB1 in hepatic metastases on PET/CT [117,118]. The tracer demonstrated excellent safety profile, low radiation dose, high metabolic stability and apparent suitability for PET diagnostic imaging of GRPR expression in oncologic patients. These promising results have opened a pathway for therapeutic applications of [^177^Lu]Lu-NeoBOMB1 in patients according to [^68^Ga]Ga-NeoBOMB1 PET findings. Hence, an international phase I/II clinical trial sponsored by the industry has been launched with the aim of identifying patients with a range of cancer types based on [^68^Ga]NeoBOMB1 PET for dose escalation treatment with [^177^Lu]Lu-NeoBOMB1, which is currently ongoing ([^177^Lu]Lu-NeoB in patients with advanced solid tumors and with [^68^Ga]Ga-NeoB lesion uptake—ClinicalTrials.gov Identifier: NCT03872778). Interestingly, a first preclinical study on the safety of PRRT with [^177^Lu]Lu-NeoBOMB1 has recently been released [119].

### 3.4. Conclusions

Based on the above, a few major conclusions can be drawn on the prospects of radiolabeled GRPR-agonists and antagonists as theranostic anticancer modalities. Radiolabeled GRPR-agonists are structurally closer to native BBN/GRP-like ligands, internalizing in target cells; however, these are mitogenic and activate the GRPR physiologically expressed in certain tissues in the body, releasing a series of side effects, and have shown unfavorably high and prolonged retention in physiological tissues [85]. Their use for radionuclide therapy was prevented due to biosafety concerns. On the other hand, radiolabeled GRPR-antagonists have undergone more extensive structural modifications than agonists becoming more “exotic” for the body and accordingly more resistant to enzymatic degradation by NEP. They neither internalize in target cells nor elicit adverse effects after iv injection, because they do not activate the receptor upon binding [13,85]. Hence, they are safer for human use with the first GRPR-antagonist motifs even being proposed as anticancer drugs [104]. They typically show faster washout from the background, even from GRPR-rich organs such as the pancreas. These qualities allow for their development into theranostic radiopharmaceuticals in GRPR-expressing tumors, such as in prostate, breast, lung cancer and gastrointestinal tumors. Multicenter clinical trials promoted by the pharmaceutical industry are expected to soon clarify the strengths and limitations of anti-GRPR-antagonist radiotheranostics in oncology.

In the case of prostate cancer theranostics, one could initially assume that the advent of radiolabeled theranostic PSMA-inhibitors in the clinic has imposed a serious challenge on the application of GRPR-antagonist radioligands [13]. However, an increasing number of studies have revealed a complementary role for both classes of compounds owing to the distinct PSMA–GRPR expression levels in various stages and types of the disease or preceding therapies, which is currently explored [120].

## 4. Gastrin-Cholecystokinin

### 4.1. Gastrin-Cholecystokinin and CCK_2_R Expression in Cancer

Gastrin and cholecystokinin (CCK) comprise a family of structurally related peptides exerting a number of shared physiological actions in the gut and the nervous system owing to a common C-terminal five amino acid fragment (Gly-Trp-Met-Asp-Phe-CO-NH_2_) [121,122,123]. Gastrin is excreted in the human antral G cells with gastrin-17 (also known as gastrin-I (GnI) or little gastrin, pGlu-Gly-Pro-Trp-Leu-Glu-Glu-Glu-Glu-Glu-Ala-Tyr-Gly-Trp-Met-Asp-Phe-NH_2_), representing the prevailing active form (95%) and gastrin-34 (or big gastrin, H-Gln-Leu-Gly-Pro-Gln-Gly-Pro-Pro-His-Leu-Val-Ala-Asp-Pro-Ser-Lys-Lys-Gln-Gly-Pro-Trp-Leu-Glu-Glu-Glu-Glu-Glu-Ala-Tyr-Gly-Trp-Met-Asp-Phe-NH_2_), also present (5%). GnI is characterized by a penta-Glu^6-10^ sequence conveying selectivity for the CCK_2_R subtype (vide infra) and better metabolic stability. Minigastrin (MG) corresponds to the GnI(5-17) fragment and MG11 to the C-terminal fragment [DGlu^10^]GnI(10-17), altogether lacking the penta-Glu sequence. Both MG and MG11 have extensively served as motifs for the development of theranostic radiopharmaceuticals [124]. Gastrin and CCK exert their actions via GPCRs located on the cell membrane of target-cells and comprising two subtypes [125,126]. The first one is the CCK subtype 1 receptor (CCK_1_R) and the second the CCK subtype 2 receptor (CCK_2_R), previously named CCK-A and CCK-B receptor, respectively. A splice variant of CCK_2_R recently discovered in human colorectal cancer cells and named CCK_2i4sv_R was shown to stimulate cell growth via a gastrin-independent mechanism [127,128]. The CCK_1_R and CCK_2_R are pharmacologically distinguished by their distinct binding affinity to gastrin and CCK ligands. Gastrin preferentially binds to CCK_2_R, whereas members of CCK with a sulphated Tyr at position 7 from the C-terminus show very high affinity for the CCK_1_R. The above two receptor subtypes differ in their tissue distribution, with the CCK_1_R being expressed in the gallbladder, gastric smooth muscles, endocrine pancreas and the peripheral nervous system, and the CCK_2_R predominantly found in the stomach and the gut mucosa.

Among the abovementioned two subtypes, the CCK_2_R is of particular relevance in oncology owing to its frequent and high-density expression in a number of human cancers [129]. Thus, medullary thyroid carcinomas (MTC) represent the most frequently CCK_2_R-expressing tumors (92%) [130], followed by astrocytomas (65%) [131] and small cell lung cancers (57%) [132], as opposed to non-small cell lung cancers showing no CCK_2_R expression [129]. Notably, all stromal ovarian cancers were found to express the CCK_2_R (100%). A number of other tumors occasionally express the CCK_2_R as well, including GEP NETs, meningiomas, endometrial and ovarian adenocarcinomas, mammary carcinomas, colon and gastric cancers [133]. High incidence of CCK_2_R expression (63%) has been documented as well in GIST, in extremely high density in most cases [83]. Based on the above, gastrin/CCK radioligands have been developed in the past decades as radiotheranostic candidates of human cancer [124,134,135].

### 4.2. Radiolabeled Gastrin/CCK Analogs in Cancer Theranostics: Major Breakthroughs

**Figure 9:**
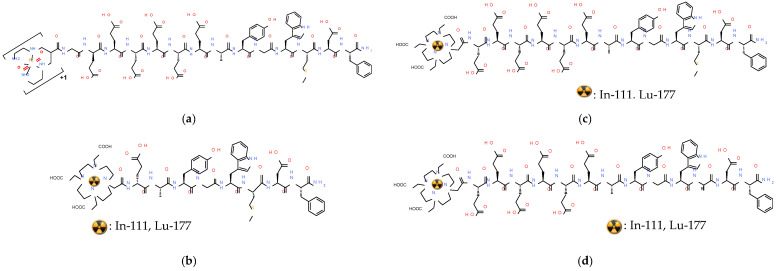
Chemical structures of (**a**) [^99m^Tc]Tc-DG2: [^99m^Tc]Tc-N_4_-Gly-DGlu-(Glu)_5_-Ala-Tyr-Gly-Trp-Met-Asp-Phe-NH_2_, (**b**) [^111^In]In-DOTA-MG0: [^111^In]In-DOTA-Dglu-(Glu)_5_-Ala-Tyr-Gly-Trp-Met-Asp-Phe-NH_2_, (**c**) [^111^In]In-DOTA-MG11: [^111^In]In-DOTA-Dglu-Ala-Tyr-Gly-Trp-Met-Asp-Phe-NH_2_, and (**d**) [^111^In]In-CP04 (Xaa: Met)/[^111^In]In-PP-F11N (Xaa: Nle): [^111^In]In-DOTA-Dglu-(Dglu)_5_-Ala-Tyr-Gly-Trp-Xaa-Asp-Phe-NH_2_.

In an analogy to the evolution of the field of somatostatin radioligands, the first CCK_2_R-targeting in humans attempted in the early 1990s involved radioiodinated GnI [124]. GnI was selected as a motif because of (i) its high selectivity and affinity for CCK_2_R, (ii) the N-terminal pyroglutamate hampering the proteolytic action of aminopeptidases and (iii) the non-sulphated Tyr^12^ allowing for radioiodination. In the first proof-of-principle study in a patient with metastatic MTC, good receptor targeting was observed in the tumor lesions and physiological organs, including the stomach and gallbladder [136]. Several other gastrin radioligands were developed thereafter, most of which were based on MG and its [DGlu^1^]MG derivative MG0 and modified at the N-terminus with DTPA, DOTA and other macrocyclic chelators to allow for stable binding of clinically relevant theranostic radiometals, with similar modifications being conducted on MG11 (Figure 9) [124,134,135]. Alternatively, coupling of HYNIC [137] or N_4_ was applied for labeling of the eminent SPECT radionuclide Tc-99m [138,139,140]. A good number of thus resulting radioligands were clinically tested mainly in MTC patients and a few examples will be discussed. Thus, [^90^Y]Y-DTPA-MG0 was investigated as a therapeutic candidate in a dose escalation study involving eight MTC participants with rapid disease progression [124,141]. All three doses applied (30 mCi/m^2^, 40 mCi/m^2^ and 50 mCi/m^2^) could induce a therapeutic effect (in six out of eight patients), related to either stable disease (four/eight patients) or partial remission (two/eight patients). However, this therapy provoked severe nephrotoxicity, especially in the patients receiving the two higher doses, thereby being disqualified [124] as an MTC therapeutic radiopharmaceutical. On the other hand, the clinical performance of [^111^In]In-DTPA-MG0 was also determined in a group of 26 MTC patients, reporting a tumor detection rate of 87% by gastrin receptor scintigraphy (GRS) [142]. GRS had a higher tumor detection rate than somatostatin receptor scintigraphy and [^18^F]FDG PET. Notably, [^111^In]In-DTPA-MG0 scintigraphy when combined with CT resulted in an impressive 96.7% detection rate of metastatic MTC. It should be noted that high renal uptake was reported in all patients. In another example, [^111^In]In-DOTA-MG11, [^111^In]In-DOTA-CCK (CCK, DAsp-Tyr-Nle-Gly-Trp-Nle-Asp-Phe-NH_2_) and [^99m^Tc]Tc-Demogastrin 2 ([^99m^Tc]Tc-DG2; DG2, [N_4_-Gly]MG0; Figure 9) were tested in a head-to-head comparison in six metastatic MTC patients for tumor visualization, renal uptake and metabolic stability [139]. According to the study outcome, [^99m^Tc]Tc-DG2 was able to visualize all known as well as previously occult lesions, and hence represents a very promising diagnostic tool in patients with evidence of MTC recurrence or metastases. The authors attributed the superior performance of [^99m^Tc]Tc-DG2 to the combination of the higher imaging qualities of Tc-99m, the high metabolic stability of the radiotracer and its unexpectedly low renal retention [139,140].

All aforementioned studies have consistently demonstrated that the penta-Glu sequence in GnI, MG and MG0 motifs is associated with high renal values of resulting radioligands, whereas its deletion leads to significant decline of kidney uptake. However, the latter benefit is compromised by the poor metabolic stability of *des*-(Glu)_5_-radioligands, negatively affecting tumor uptake [139,143,144]. To overcome this handicap, a number of methodologies have been proposed. In an innovative approach, *des*-(Glu)_5_-radioligands have been employed together with protease inhibitors and especially inhibitors of NEP, such as PA, or thiorphan. This methodology was found to be particularly effective to enhance the tumor uptake of *des*-(Glu)_5_-radioligands without unfavorably increasing radioactivity levels in the kidneys, resulting in impressive increases of tumor-to-kidney values in animal models [100,144,145]. Notably, this approach could be recently confirmed in four MTC patients receiving [^111^In]In-DOTA-MG11 following oral administration of Hidrasec^®^. The latter is registered as an anti-diarrhea agent containing the prodrug racecadotril. When taken per os racecadotril releases in vivo the potent NEP-inhibitor thiorphan [101,145]. Combination of [^111^In]In-DOTA-MG11 with racecadotril resulted in a significant increase of radiolabel uptake in MTC lesions compared to controls and additionally visualized a previously unknown lesion in one of the patients [146]. Further studies are ongoing to optimize this effective and safe methodology in the clinic.

At the same time, structural interventions on GnI, MG0 and MG11 motifs have been evaluated in a concerted European research project. In this respect, 12 DOTA-derivatized analogs (2 of which were CCK-based) were labeled with In-111 and compared head-to-head in cells and animal models [143]. Firstly, this study confirmed all previously established conclusions, related to the impact of the penta-Glu chain. Moreover, 3 out of the 12 analogs, each one representing a distinct structural modification route, displayed the best pharmacokinetic profiles. The first successful modification concerned the substitution of the penta-Glu^6-10^ chain in [^111^In]In-DOTA-MG0 by penta-Dglu with the respective [^111^In]In-PP-F11 (or else [^111^In]In-CP04) displaying comparable tumor uptake but a 90% reduced kidney uptake. The second modification concerned the bivalent [^111^In]In-MGD5 (MGD5, DOTA-Gly-Ser-Cys-(Glu-Ala-Tyr-Gly-Trp-Nle-Asp-Phe-NH_2_)_2_) which outperformed its monomer [^111^In]In-APH070 version (APH070, DOTA-His-His-Glu-Ala-Tyr-Gly-Trp-Nle-Asp-Phe-NH_2_). The third involved a cyclization, yielding [^111^In]In-DOTA-cyclo-MG1 (cyclo-MG1, Dglu-(Ala-Tyr)-Dlys-Trp-Met-Asp-Phe-NH_2_ (cyclo Dglu-Dlys)). These structural changes were further elaborated yielding new interesting analogs, with a few currently undergoing clinical evaluation as theranostic candidates in the management of MTC and possibly other CCK_2_R-expressing human cancers [135].

Thus, CP04 (or PP-F11) was developed into a kit for the preparation of the theranostic [^111^In]In/[^177^Lu]Lu-CP04 pair within a European cooperation to conduct a translational Phase I multi-center clinical trial in 16 metastatic MTC patients (www.clinicaltrials gov (NCT03246659) and EudraCT (2015-000805-38)), using two different peptides masses, 10 and 50 μg, and co-infusion of the kidney protection agent gelofusine in a randomized fashion [147,148,149]. Results of the study were recently reported, showing that [^111^In]In-CP04 slightly outperformed the conventional imaging methods used as reference (detection rates: 81% over 75%). Preliminary dosimetry studies revealed the highest dose to urinary bladder, followed by the kidneys and stomach wall. The effective dose (7 mSv for 200 MBq) of [^111^In]In-CP04 was found to be comparable for the two peptide amounts. Gelofusine reduced the dose to the kidneys by 53%, resulting in the organ-absorbed dose of 0.044 ± 0.019 mSv/MBq, with the projected absorbed dose to the kidneys for [^177^Lu]Lu-CP04 estimated at 0.9 ± 0.4 Gy/7.4 GBq. This study showed that the [^111^In]In/[^177^Lu]Lu-CP04 pair is a promising agent in the management of human MTC and more dedicated studies are warranted to fully establish its clinical value [149]. Interestingly, in a prospective, phase 0 single-center study (ClinicalTrials.gov: NCT02088645) [^177^Lu]Lu-PP-F11N, the Nle^11^ version of [^177^Lu]Lu-CP04, was evaluated in six consecutive patients for its suitability in the treatment of MTC applying advanced 3-dimensional dosimetry [150]. Patients received two injections of approximately 1 GBq [^177^Lu]Lu-PP-F11N (corresponding to ~80 μg peptide-conjugate) with or without nephroprotection with gelofusine. This study showed that [^177^Lu]Lu-PP-F11N accumulated specifically in MTC at a dose sufficient for therapy, displaying low kidney and bone marrow radiation dose and the stomach most likely representing the dose-limiting organ. Further clinical studies will determine the maximum tolerated dose and the efficacy of [^177^Lu]Lu-PP-F11N in the treatment of MTC and other CCK_2_R-expressing human tumors.

**Figure 10:**
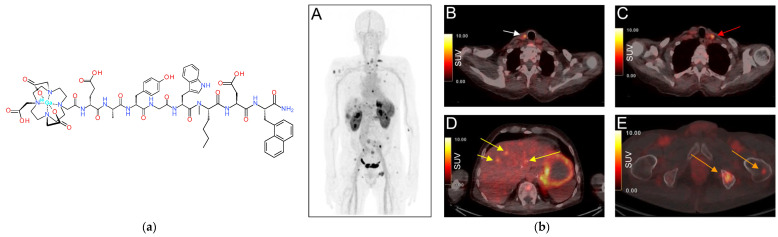
(**a**) Chemical structure of [^68^Ga]Ga-MGS5: [^68^Ga]Ga DOTA-DGlu-Ala-Tyr-Gly-Trp-(N-Me)Nle-Asp-1-Nal-NH_2_, (**b**) MIP (**A**) and axial fused PET/CT with [^68^Ga]Ga-MGS5 at 1 h pi in a metastatic MTC patient with local recurrence on the right paratracheal region (**B**) white arrow, left cervical lymph node metastasis (**C**) red arrow, multiple hepatic metastases (**D**) yellow arrows, and metastases in the left iliac bone and left femur (**E**) orange arrows. This research was originally published in *JNM* 2023 ([151]; https://jnm.snmjournals.org/content/early/2023/01/19/jnumed.122.264977).

As a last example, the first human data from a comparative study of [^68^Ga]Ga-MGS5 (MGS5: DOTA-Dglu-Ala-Tyr-Gly-Trp-(N-Me)Nle-Asp-1-Nal-NH_2_; Figure 10) and [^18^F]F-DOPA on PET/CT in a recurrent MTC patient were reported [151]. The two tracers detected several common but also different lesions, providing complementary information. In a subsequent study involving six patients with advanced MTC were imaged with [^68^Ga]Ga-MGS5 PET/CT. A total of 87 lesions with increased radiotracer uptake considered malignant was detected with higher lesion to background ratios achieved at 2 h pi, revealing the excellent prospects of [^68^Ga]Ga-MGS5 PET/CT in the detection of local recurrence and metastases in patients with advanced MTC.

### 4.3. Biosafety Problems: Development of Radiolabeled CCK_2_R-Antagonists Today

The abovementioned clinical studies of radiolabeled CCK_2_R-agonists based on gastrin have brought to light a series of adverse effects following the ligand-induced CCK_2_R-activation in analogy to those induced during GRPR-activation by potent BBN/GRP radioligands. These effects resemble those elicited during the provocative pentagastrin (Boc-βAla-Trp-Met-Asp-Phe-NH_2_) test, including nausea, abdominal cramps, tachycardia and flush [152]. The severity of these effects depended on the amount of administered peptide, its CCK_2_R affinity and potency, the metabolic stability and other factors. This fact is expected to restrict the doses injected in patients, especially during radionuclide therapy. On the other hand, the accumulation of radioactivity in CCK_2_R-rich organs, such as the stomach wall, may have implications as well during therapy. Indeed, dosimetric calculations using radiolabeled CCK_2_R-agonists have indicated the gastric wall, along with the kidneys, as the dose-limiting organ [149,150,151].

**Figure 11:**
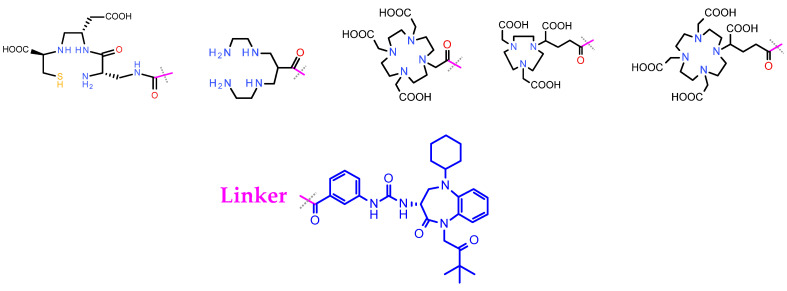
Chemical structures of Z360 (nastorazepide) analogs with different chelators coupled to its free carboxy group via a linker for labeling with theranostic radiometals, such as Tc-99m, Ga-67/68, In-111 and Lu-177 and showing antagonistic properties at the CCK_2_R.

An elegant way to evade these problems is the application of radiolabeled CCK_2_R-antagonists for the theranostic management of MTC and other human tumors. Interestingly, CCK_2_R-antagonists developed by pharmaceutical industry may provide useful motifs for the development of the respective radiometal-carrying versions [153]. It should be noted, however, that such antagonists are non-peptidic benzodiazepine analogs and the design of the respective radioligands can be challenging. Accordingly, the field of radiolabeled theranostic CCK_2_R-antagonists is still in its infancy, with only a few tracers developed and preclinically tested. Most efforts have used nastorazepide or else Z-360 (3-[[1-cyclohexyl-5-(3,3-dimethyl-2-oxobutyl)-4-oxo-2,3-dihydro-1,5-benzo-diazepin-3-yl]-carbamoylamino-benzoic acid) as a motif, with a free carboxy group amenable for coupling of suitable chelators either directly or via different linkers [154,155]. Coupling of N_3_S [156] or N_4_ [14] chelators has allowed for labeling with Tc-99m for SPECT imaging, whereas coupling of DOTA, DOTAGA and NODAGA allows the stable coordination of theranostic trivalent radiometals for SPECT or PET imaging and for radionuclide therapy, as presented in Figure 11 [157,158,159]. Preclinical results thus far acquired are quite promising and further studies are warranted to provide evidence on the validity of this option.

### 4.4. Conclusions

The field of CCK_2_R-directed theranostics has been thriving in the past two decades with a number of improved gastrin analogs tested in the clinic. Diagnosis of MTC, either localized, recurrent or disseminated, applying SPECT or PET combined with CT, has shown the first convincing results with several efforts toward the initiation of therapeutic approaches. The latter can be applied as monotherapies, but adjuvant schemes may turn out to be more effective and less toxic. Combination with kidney protection regimens, such as the infusion of gelofusine, have often shown success in increasing tumor-to-kidney ratios also in patients [149]. Likewise, administration of NEP inhibitors was found to be a promising approach to enhance tumor localization of biodegradable analogs, such as [^111^In]In-MG11, in MTC patients with good prospects of improving the efficacy of the respective radiotherapeutics in lower administered and less toxic doses [146]. Combination of radiotherapeutics and mTOR inhibitors preclinically tested for [^177^Lu]Lu-PP-F11N in mice, has resulted in improvement of tumor uptake, presumably via the CCK_2_R-upregulation in tumor cells, offering hopes for a successful translation in the clinic [160]. Likewise, the application of alpha therapy, recently presented for [^225^Ac]Ac-PP-F11N in mice demonstrated dose-dependent inhibition of tumor growth and extended mean survival time, without apparent toxicity. The histological analysis of kidney and stomach indicated no severe adverse effects after administration, raising expectations for clinical translation in future [161].

In this respect, the advent of the next generation of CCK_2_R-antagonist based theranostic radioligands, such as benzodiazepine derived agents, may provide additional and unique advantages. These include a higher resistance to proteolytic enzymes as opposed to peptide radioligands, the lack of side effects provoked by receptor-activation and a faster background clearance, typical for radiolabeled antagonists. These attributes will allow for the administration of higher doses during radiotherapy and higher therapeutic index. Such exciting prospects will be addressed in the near future to the benefit of patients with MTC and other CCK_2_R-related cancers.

## 5. Exendin—Glucagon-like Peptide 1 Receptor

### 5.1. Exendin and Glucagon-like Peptide 1 Receptor in Insulinoma

A high density of the glucagon-like peptide 1 receptor (GLP-1R) expression was identified on frozen insulinoma biopsy specimens [162,163], indicating GLP-1R as a potential target for radiotheranostics [164]. Insulinomas are the most common type of functioning pancreatic NETs, originating from the insulin-producing beta cells in the islets of Langerhans. In the majority of cases, insulinomas are benign and yet the symptoms (mainly hyperinsulinemic hypoglycemia due to secretion of insulin by the lesion) are severe and require fast and accurate treatment [165,166]. For localized disease, resection of the lesion or partial pancreatectomy are still the only curative options. In view of the small size (83% ≤ 2 cm) of most lesions, 5–10% of them are missed on conventional imaging, such as CT, MRI, or endoscopic ultrasonography (EUS). Furthermore, 10–27% of the lesions are not identified intraoperatively [165,167]. In order to guide the surgical procedure and to minimize unnecessary surgical interventions, precise preoperative localization of the lesion is of pivotal importance. Therefore, radiolabeled analogs of the GLP-1R natural ligand, GLP-1 (H-His-Ala-Glu-Gly-Thr-Phe-Thr-Ser-Asp-Val-Ser-Ser-Tyr-Leu-Glu-Gly-Gln-Ala-Ala-Lys-Glu-Phe-Ile-Ala-Trp-Leu-Val-Lys-Gly-Arg-NH_2_), have been recently developed. It should be noted that GLP-1 has a very short biological half-life; thus, several stable analogs of GLP-1 had to be developed. The agonist exendin (Ex, H-His-Gly-Glu-Gly-Thr-Phe-Thr-Ser-Asp-Leu-Ser-Lys-Gln-Met-Glu-Glu-Glu-Ala-Val-Arg-Leu-Phe-Ile-Glu-Trp-Leu-Lys-Asn-Gly-Gly-Pro-Ser-Ser-Gly-Ala-Pro-Pro-Pro-Ser-NH_2_), isolated from the saliva of the *Heloderma suspectum* (Gila monster), is a stable analog of GLP-1 with high binding affinity to GLP-1R [168]. Nowadays, visualization of benign insulinomas with PET/CT by GLP-1R-directed radioligands is an established and high-sensitivity diagnostic technique [166,169].

### 5.2. Radiolabeled Exendin Analogs in Theranostics: Problems in Clinical Translation

#### 5.2.1. PRRT

The only curative treatment for insulinomas is surgical resection, a treatment still associated with major risks despite the currently applied sensitive nuclear imaging techniques, which have considerably improved lesion localization. Notably, morbidity rates of 33–56% and mortality rates up to 6% are still reported for this treatment [170]. Furthermore, surgical intervention is not a valid option for patients when the lesion is close to a large vessel or the pancreatic duct or when multiple lesions are present [171]. Hence, alternative strategies to treat insulinomas are urgently needed [172]. Thus far, very few therapeutic GLP-1R-directed radioligands based on exendin are reported. The first study in this direction explored the therapeutic potency of [Lys^40^(Ahx-[^111^In]In-DTPA)NH_2_]Ex-4 based on the Auger electrons of In-111. The agent was successful in reducing tumor burden in mice, but induced severe nephrotoxicity eventually disqualifying this type of treatment [173]. In another dosimetry study, ([^177^Lu]Lu-DO3A-VS-Cys^40^]Ex-4 was evaluated as a therapeutic agent in a rat model. Extrapolation of rat to human data, estimated a maximal activity dose of 3.8 GBq to ensure a kidney dose below the maximal tolerated dose of 23 Gy. However, the allowed activity dose of 3.8 GBq was considered insufficient for therapeutic efficacy [174]. It is evident that the application of exendin-based radioligands for PRRT of insulinomas patients has been hampered by the high kidney accumulation. Another important factor hindering the completion of a well-controlled clinical study on the efficacy of PRRT with radiolabeled exendin is the low incidence of insulinomas.

Several strategies were pursued to overcome the problem of excessive kidney uptake of radiolabeled exendin. In a first approach, a cleavable linker was introduced between exendin and the radiometal-chelate aiming to exploit the proteolytic action of enzymes at the kidney brush border membrane to cleave the radiometal-chelate from the peptide, thereby promoting the fast shifting of radioactivity from the kidneys into urine. Indeed, a significant reduction in renal uptake of ([^68^Ga]Ga-NOTA-MVK-Cys^40^-Leu^14^]Ex-4, was achieved without negatively affecting tumor uptake in mice [175]. In another interesting approach, an albumin-binding moiety was added to exendin-4 to increase circulation times. As a result, reduction in kidney uptake was achieved in a mouse model without decreasing tumor values [176,177]. In a clinical study, gelofusine was used to reduce the renal uptake of ^111^In-labeled exendin-4. The data acquired from human planar scintigraphy was converted to Lu-177 and the maximum activity of ^177^Lu-labeled exendin-4 allowed to be injected in the gelofusine regimen was estimated to increase by almost 22% [178]. Although PRRT with radiolabeled exendin is not an option yet, several recent studies have been successful in reducing renal uptake and consequently radioactive doses to the kidneys. These findings are very encouraging and may eventually allow for PRRT of insulinoma with radiolabeled exendin in future.

#### 5.2.2. PDT

**Figure 12:**
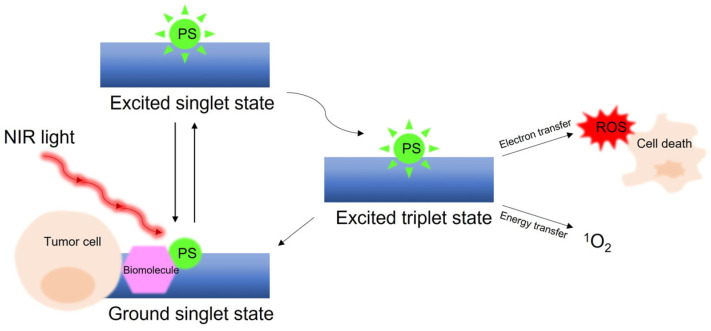
A schematic Jablonski diagram showing targeted photodynamic therapy (tPDT). The method is based on a photosensitizer (PS) attached to a molecule selective for a certain target. The PS is activated by light of a specific wavelength (preferably in the near infrared range, NIR) to a higher-energy state, upon which energy is transferred from the activated PS to surrounding oxygen to form reactive oxygen species (ROS). These can induce irreversible damage to a cell directly or to the tumor-associated vasculature, leading to cell death.

An alternative therapy to PRRT would rather aim at eliminating life-threatening symptoms of insulinoma (like hyperinsulinemic hypoglycemia) than eradicating the tumor. By depleting part of the tumor, the insulin production of the lesion could theoretically be lowered to such an extent that the glucose homeostasis in the patient is normalized. In this respect, receptor targeted photodynamic therapy (rtPDT) represents a suitable therapy (Figure 12) [179,180,181]. A major advantage of this technique is the absence of kidney toxicity. This kind of minimally invasive therapy has been widely studied in oncology with a few studies investigating the use of PDT on insulinoma cells [182] or in pancreatic cancers [183].

Interestingly, PDT with exendin-4 coupled to the photosensitizer IRDye700DX was recently reported to repress tumor growth and improve median survival in BALB/c nude mice bearing GLP-1R expressing tumors [184]. As the study was conducted in a tumor model with non-physiological GLP-1R expression, the efficacy of the treatment needs to be confirmed in a model with GLP-1R expression comparable to human insulinomas. Of particular interest is the high dose of exendin-4 used in the PDT study required for an optimal therapeutic effect [185]. How the above data on the preclinical application of PDT with exendin-4-IRDye700DX translate in the human situation needs to be further investigated. One can hypothesize that the amount of exendin that has to be delivered to a patient lesion and thus has to be administered to the patient is higher compared with current clinical practice with radiolabeled exendin. Several studies describe the adverse events following exendin administration in humans, such as nausea, vomiting and hypoglycemic episodes (in cases not receiving glucose infusion) [186]. Thus, it is rational to expect that the advent of GLP-1R-antagonists will successfully address these problems.

### 5.3. From Radiolabeled GLP-1R-Agonists to Antagonists

Ex(9-39) is a truncated derivative of the GLP-1R-agonist exendin, lacking the N-terminal sequence inducing receptor internalization and activation, and thus representing a true GLP-1R-antagonist [187,188]. Several studies have investigated whether the pharmacokinetics of the radiolabeled Ex(9-39) would be superior to radiolabeled exendin-4. If so, the side effects provoked by agonist activation of the GLP-1R, such as nausea, vomiting and hypoglycemia, could be avoided. A head-to-head comparison of [Lys^40^([^111^In]In-DTPA)]Ex-4 and [Lys^40^([^111^In]In-DTPA)]Ex(9-39) revealed similar affinity for the GLP-1R and the same number of binding sites; however, the receptor antagonist displayed disappointingly low tumor uptake in rat insulinomas [189]. Conjugation at either Lys^40^ or Lys^27^ equally led to low uptake in rat insulinomas for [Lys^27^([^68^Ga]Ga-NODAGA)]Ex(9-39) and [Lys^40^(([^68^Ga]Ga-NODAGA)]Ex(9-39) [190]. Furthermore, Ex(9-39) harboring BnDTPA (2-(4-aminobenzyl)diethylenetriaminepentaacetic acid) at position 12 (Lys^12^(BnDTPA)]Ex(9-39) showed loss of binding affinity to GLP-1R. On the other hand, BnDTPA-Ex(9-39) (BnDTPA coupled at the N-terminus) showed a higher tumor uptake than [Lys^40^([^111^In]In-DTPA)]Ex(9-39) [189,191], a result most probably generated by the much lower peptide dose used in the latter study (below the GLP-1R saturability range of the experimental tumor). The choice of the amino acid for radioiodination of Ex(9-39) was found to be important for the profile of resulting radioligand. Thus, [^125^I]I-Ex(9–39) via the Bolton–Hunter (BH) reaction (3-(4-Hydroxyphenyl)propionic acid N-hydroxysuccinimide ester) on Lys^27^ presented excellent binding in rat and Rip1Tag2 mouse insulinomas, but not in human insulinomas [15,190]. For an easier translation in a clinical setting, direct iodination methods were investigated. Thus, [Nle^14^,[^125^I]I-Tyr^40^]Ex(9–39) recognized more binding sites compared to [Nle^14^,[^125^I]I-Tyr^40^]Ex-4, but the antagonist achieved lower uptake in rat insulinomas than its agonist counterpart, presumably due its lower affinity and lack of internalization [192]. It is noteworthy that most studies were conducted in rodent insulinomas, although the receptor affinities of different Ex(9-39) derivatives were reported to be species-dependent. According to these lines, by radioiodination of Ex(9-39) at Lys^4^ solely rat insulinomas could be identified, whereas at position Lys^19^, both rat and human insulinomas could be recognized by the respective radioligands [193]. Despite these efforts, a GLP-1R-antagonist radioligand based on Ex(9-39) has not yet made it to clinical practice as a diagnostic or therapeutic agent due to low tumor uptake, rapid washout from tumor-sites, or lack of affinity to the human GLP-1R.

### 5.4. Conclusions

The perspectives of PRRT of insulinomas with a radiolabeled agonist remain remote today due to high kidney uptake and adverse effects elicited by GLP-1R activation. On the other hand, the clinical value of radiolabeled GLP-1R-antagonists based on Ex(9-39) remains controversial as well, with most researchers disputing the applicability of related radioligands in insulinoma imaging, let alone treatment. The position to which a chelator is conjugated or radioiodine is introduced in the Ex(9-39) peptide chain was found to be important in determining the affinity to the human GLP-1R. Furthermore, validation of the compounds in a proper model is crucial, as human insulinomas appear to have a lower receptor density than rodent insulinoma models [163]. Finally, although radioiodinated Ex(9-39) seems to be superior to exendin-4 in terms of kidney uptake, it proved to be less suitable for the treatment of insulinoma, due to low tumor uptake, release and rapid washout of radioiodine in the body.

It would be interesting to hypothesize on the design of a GLP-1R radioligand combining the best traits of an agonist with those of an antagonist, in other words capable to internalize without inducing adverse pharmacological effects after binding to GLP-1R. Concerning PDT requiring high photosensitizer amounts to reach the GLP-1R on the tumor, the use of an antagonist is the rational method to follow. To elaborate on this aspect, a possible way to enhance the lesion uptake of the radiolabeled antagonist is the use of cell-penetrating peptides (CPPs). Notably, [Lys^46^[^111^In]In-DTPA]Ex(9-39)-Pen, where penetratin (H-Arg-Gln-Ile-Lys-Ile-Trp-Phe-Gln-Asn-Arg-Arg-Met-Lys-Trp-Lys-Lys-NH_2_) serves as the CPP, demonstrated a higher uptake in rat insulinomas compared to [Lys^31^[^111^In]In-DTPA]Ex(9-39) in a proof-of-concept study. Nevertheless, the issue of receptor activation needs to be further investigated [194].

It is reasonable to conclude that there is still hope on the horizon with regards of PRRT of insulinoma using radiolabeled exendin. Whether it will be an agonist with lower renal accumulation or an antagonist with increased lesion uptake needs to be elucidated.

## 6. Concluding Remarks

Based on the above, it is evident that the field of peptide radiopharmaceuticals for use in cancer theranostics has undeniably gone through a remarkable growth over the past two decades. Following the clinical advent of the first approved peptide radiopharmaceutical OctreoScan^®^ in the diagnostic imaging of NETs, somatostatin analogs with improved profiles have also reached the market and eventually our patients. A recent example is the radiotheranostic pair [^68^Ga]Ga/[^177^Lu]Lu-DOTA-TATE, which has opened the way for further breakthroughs in nuclear oncology. These do not only concern broadening the spectrum of human tumors to encompass cancers expressing other GPCR targets beyond the SST_2_R. Most importantly, newest technological developments in imaging modalities (e.g., the state-of-the-art imaging instrumentation, such as the total-body PET/CT scanner) [195], access to new therapeutic radionuclides (e.g., the alpha emitters Ac-225, or Pb-212, and the Auger electron/beta emitters, such as Tb-161) [196,197], as well as innovative adjuvant therapeutic schemes (e.g., radiosensitizers, or Poly(ADP-ribose) polymerase-1 (PARP) inhibitors, mTOR inhibitors, or other means) [69,70,160], represent a solid background for exciting new advances in near future.

In this respect, GPCR-antagonist-based radioligands play an increasingly important role. While much progress has already been achieved in the field of somatostatin receptor antagonists, in view of the higher number of binding sites on tumor lesions available to antagonists and the excellent pharmacokinetics of related radioligands, this change in paradigm infiltrates toward other peptide families. In other GPCR types, the shift to antagonists becomes even more essential due to biosafety concerns, given that peptide ligands with agonistic behavior, e.g., at the GRPR, CCK_2_R and GLP-1R, elicit adverse effects after receptor activation following systemic administration in humans. The advent of radiolabeled GRPR-antagonists has enabled the performance of therapy clinical studies, providing hopes for safe and effective PRRT in a broader spectrum of frequently occurring cancers. At this moment, however, this option is in its early stages in the case of CCK_2_R and GLP-1R radioligands. Many open questions and challenges need to be rigorously addressed prior to a broader application of radiolabeled receptor antagonists. For example, it is still unknown if and to what extent non-internalizing receptor antagonists radiolabeled with alpha or even Auger electron emitters can induce apoptosis of cancer cells. Moreover, it is very interesting to better understand the mechanism(s) underlying the faster background clearance of radiolabeled antagonists, even from tissues physiologically expressing their cognate GPCR, as opposed to their longer retention in tumor sites. There is no doubt that research in the field is thriving and exciting new outcomes will most likely reach us in near future.

## Figures and Tables

**Table 1:** Peptide ligands and their cognate GPCR-targets overexpressed in human tumors.

Peptide	Receptor	Tumor Expression
Somatostatin	SST_1_R, SST_2_R, SST_3_R, SST_4_R, SST_5_R	NET, NHL, melanoma, BC, MTC, SCLC
Bombesin/GRP	BB_1_R/NMBR, BB_2_R/GRPR, BB_3_R	PC, BC, SCLC, colorectal cancer, glioblastoma, gastrinoma, GIST
CCK/gastrin	CCK_1_R, CCK_2_R	MTC, SCLC, astrocytoma, stromal ovarian cancer, GIST
GLP/Exendin	GLP-1R	insulinoma
Neurotensin	NTS1R, NTS2R, NTS3R	SCLC, PDAC, Ewing sarcoma, meningioma, astrocytoma
Substance P	NK_1_R, NK_2_R, NK_3_R	Glioblastoma, astrocytoma, SCLC, MTC, BC
NPY	NPY1R, NPY2R, NPY4, NPY5	PC, renal cell carcinoma, ovarian adenocarcinoma, neuroblastoma, paraganglioma, GIST
VIP	VPAC1R, VPAC2R	SCLC, colorectal cancer, BC, gastrinoma, PC
α-MSH	MC_1-5_R	Melanoma

NET: neuroendocrine tumor, NHL: non-Hodgkin’s lymphoma, BC; breast cancer, MTC: medullary thyroid carcinoma, SCLC: small cell lung cancer, GRP: gastrin releasing peptide, PC: prostate cancer, GIST: gastrointestinal stromal tumors, GLP: glucagon-like peptide, GLP-1R: glucagon-like peptide-1 receptor, PDAC: pancreatic ductal adenocarcinoma, NPY: neuropeptide Y, VIP: vasoactive intestinal peptide, α-MSH: α-melanocyte-stimulating hormone.

## Data Availability

Data sharing not applicable.
